# Bone Cell Exosomes and Emerging Strategies in Bone Engineering

**DOI:** 10.3390/biomedicines10040767

**Published:** 2022-03-24

**Authors:** Sanjana Vig, Maria Helena Fernandes

**Affiliations:** 1 Faculdade de Medicina Dentaria, Universidade do Porto, Rua Dr. Manuel Pereira da Silva, 4200-393 Porto, Portugal; 2 LAQV/REQUIMTE, University of Porto, 4160-007 Porto, Portugal

**Keywords:** exosomes, osteoclasts, osteoblasts, osteocytes, mesenchymal stem cells, endothelial cells, immune cells, bone tissue engineering

## Abstract

Bone tissue remodeling is a highly regulated process balancing bone formation and resorption through complex cellular crosstalk between resident bone and microenvironment cells. This cellular communication is mediated by direct cell and cell–matrix contact, autocrine, endocrine, and paracrine receptor mediated mechanisms such as local soluble signaling molecules and extracellular vesicles including nanometer sized exosomes. An impairment in this balanced process leads to development of pathological conditions. Bone tissue engineering is an emerging interdisciplinary field with potential to address bone defects and disorders by synthesizing three-dimensional bone substitutes embedded with cells for clinical implantation. However, current cell-based therapeutic approaches have faced hurdles due to safety and ethical concerns, challenging their clinical translation. Recent studies on exosome-regulated bone homeostasis and regeneration have gained interest as prospective cell free therapy in conjugation with tissue engineered bone grafts. However, exosome research is still in its nascent stages of bone tissue engineering. In this review, we specifically describe the role of exosomes secreted by cells within bone microenvironment such as osteoblasts, osteocytes, osteoclasts, mesenchymal stem cell cells, immune cells, endothelial cells, and even tumor cells during bone homeostasis and crosstalk. We also review exosome-based osteoinductive functionalization strategies for various bone-based biomaterials such as ceramics, polymers, and metals in bone tissue engineering. We further highlight biomaterials as carrier agents for exosome delivery to bone defect sites and, finally, the influence of various biomaterials in modulation of cell exosome secretome.

## 1. Introduction

Bone tissue remodeling involves a balance between bone formation and bone resorption process mediated by several signaling mechanisms [1] including direct cell–cell contact [2], cell–matrix interaction [3], autocrine, endocrine (hormones) [4], and paracrine factors (soluble molecules, vesicles, and growth factors) between bone cells [5,6,7] and their microenvironment. There is growing evidence now that the cells involved in bone remodeling also secrete extracellular vesicles (EVs), which influence bone cell crosstalk by regulating bone cell proliferation, differentiation, migration, apoptosis, and metabolism through various signaling pathways [8,9,10]. Extracellular vesicles are a heterogeneous population of phospholipid bilayer membrane-bound vesicles classified into exosomes (40–120 nm), microvesicles (50–1000 nm), and apoptotic bodies (500–2000 nm) depending on their origin, size, and function [11,12]. While microvesicles and apoptotic bodies are shed from the plasma membrane of cells, exosomes are from endocytic origin [13]. Among these extracellular vesicles, exosomes have been gaining attraction due to growing evidence linking their role in bone physiology and pathology [14,15,16,17]. 

Exosomes are nanometer-sized extracellular vesicles of endocytic origin that are secreted in the extracellular matrix by various cells for paracrine signaling and communication [18,19]. Exosomes contain a unique cargo of proteins, lipids, and nucleic acids (DNA, mRNA, microRNAs, and non-coding RNAs), which can be transferred to the recipient cells to mediate intercellular communication [20]. Due to their lipophilic nature, exosomes have the potential to be internalized into target cells by various mechanisms such as receptor mediated endocytosis [21], lipid raft-mediated endocytosis [22], clathrin-mediated endocytosis [23], caveolin-mediated endocytosis [24], phagocytosis, and micropinocytosis to mediate specific responses [25]. Upon internalization into recipient cells, exosomes release their cargo contents and regulate the gene expression, thus, influencing their commitment, differentiation, and fate [26]. While exosomes of all cells contain similar marker proteins (tetraspanin molecules CD9, CD63, and CD81, and tumor susceptibility gene 101, Alix protein, and heat shock protein 70) due to their endosomal origin and biogenesis [27], exosomes also express membrane proteins specific to their parent cells [28]. Exosomes have been demonstrated to play a crucial role in bone tissue homeostasis depending on their origin in resident bone cells or in microenvironment [29,30].

Bone homeostasis is highly regulated with coordinated crosstalk between osteoblasts, osteocytes, and osteoclasts, which interact with their microenvironment [31,32,33,34,35]. The impairment of this bone remodeling process leads to development of bone disorders and diseases [36]. An emerging research area to address these conditions is bone tissue engineering, involving amalgamation of fields of material sciences and bone cell biology [37,38]. Several bone specific biomaterials are being investigated as suitable substitutes for graft transplantations depending on various physiochemical and biological characteristics such as mechanical properties, porosity, biocompatibility, osseointegration, etc. [39,40,41,42]. The most popular materials studied include metals, ceramics, polymers, and hydrogel systems [43,44]. Several studies have been performed on these fabricated scaffolds embedded with bone specific cells and progenitors to demonstrate potential of these materials to support proliferation and differentiation of cells [45,46,47,48]. However, cell-based therapy has met with several issues during clinical application such as concerns of safety, immune reactions, ethical approvals, exorbitant costs, and long expansion time [49,50]. Recently, with evidence of exosomes mediated regulation of bone homeostasis, there has been interest in studying exosomes in context of bone tissue engineering [51,52,53,54,55]. Exosomes are now being perceived as a cell free therapy for conjugation on biomaterials for functionalization and therapeutic application [56,57]. Moreover, preliminary studies have also indicated to the modulation of exosome cargo during cell interaction with biomaterials [58]. Research is also being conducted on alternative techniques for generating exosomes at higher yields and also synthesizing exosome mimetics artificially through extrusion approach [59]. Exosome research on tissue engineered bone biomaterials is still in its nascent stages requiring detailed studies [60,61]. In this review, we will provide a comprehensive discussion on the present literature focusing on exosome-mediated bone tissue remodeling under normal and 3D bone tissue states.

## 2. Bone Tissue Remodeling and Crosstalk

**Figure 1:**
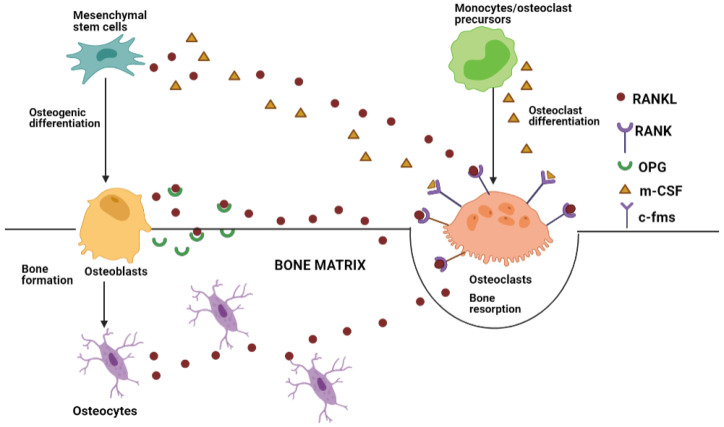
Schematic representation of role of RANKL/RANK/OPG signaling in Bone homeostasis. Bone formation initiates with osteogenic differentiation of mesenchymal stem cells into osteoblasts. A sub population of these osteoblasts are terminally differentiated into osteocytes, which embed inside the bone matrix. Specific matrix sensing by the osteocytes triggers recruitment of osteoclast precursors (monocytes/macrophages), which differentiate into osteoclasts. During early osteoclast differentiation, MSCs and the precursor cells secrete M-CSF, which binds to c-fms receptor on the osteoclasts. This process is also coupled with release of RANKL from MSCs, osteoblasts, and osteocytes, which binds to RANK receptor on osteoclasts. Additionally, osteoblasts also secrete OPG inhibitor for competitive inhibition of RANKL to counter the bone resorption process.

Bone is a highly mineralized connective tissue consisting of resident cells such as osteoclasts, osteoblasts, osteocytes, bone lining cells, and an organic and inorganic extracellular matrix [62]. Bone homeostasis is a dynamic process regulated by the resident bone cells as well as the interacting cells of the microenvironment such as endothelial cells, immune cells and stromal cells [63]. While osteoblasts are responsible for bone formation, osteoclasts lead to bone resorption to remove matrix degradation products. The impairment of this balance is associated with development of bone pathologies such as osteoporosis, arthritis, osteosarcoma, etc. [64]. Bone formation origins from the osteogenic commitment of mesenchymal stem cells, which differentiate into osteoblasts that constitute about 4–6% of the resident cells of the bone [65,66]. Osteoblasts are responsible for synthesis of the bone matrix in a two-step process. The first step involves formation of organic matrix consisting of collagen proteins (mostly Type 1 collagen), glycoproteins such as osteonectin, osteopontin, bone sialoprotein, and proteoglycans such as decorin and hyaluronan [67]. The second step involves mineralization with inorganic ions such as calcium and phosphate, which nucleate to form the hydroxyapatite crystals [68]. Differentiation of mesenchymal stem cells into osteoblasts involves activation of two major signaling pathways—Bone morphogenic protein (BMPs)/transforming growth factor B as well as the Wingless type Wnt/b catenin pathway wherein the master gene, Runt-related transcription factor 2 (*RUNX2*), is upregulated [69,70]. A sub population of these osteoblasts terminally differentiates into osteocytes that embed into the bone matrix to form the osteocytes that are the most abundant cells in bone tissue (90–95%) [71]. Osteocytes are highly mechanosensitive cells and act as sensors, which help body to adapt to external pressures and mechanical forces [72]. The process of maturation of osteocytes involves downregulation of osteoblast genes and activation of osteocyte specific proteins such as dentine matrix protein (DMP1) and sclerostin [73]. Osteocyte apoptosis triggers the recruitment of the bone-resorbing cells, osteoclasts, which initiate the remodeling process [74]. Osteoclasts are multinucleated cells originating from the hematopoietic progenitors in the bone marrow, which also form monocytes in the peripheral blood and tissue macrophages [75]. A cellular crosstalk occurs between the bone cells to balance the resorption and formation process as illustrated in Figure 1. During bone resorption process, macrophage colony stimulating factor (M-CSF) is secreted by the progenitor mesenchymal stem cells as well as osteoblasts and binds to its receptor (c-fms) on osteoclast precursor cells to stimulate their proliferation. This process is coupled with binding of Receptor activator of nuclear factor kb (RANK) ligand (expressed on osteoblasts, osteocytes, and stromal cells) on the RANK receptor present on osteoclast precursor cells [76]. This positively regulates expression of osteoclast specific proteins such as Tartrate resistant acid phosphatase (TRAP), cathepsin K, Nuclear factor of activated T-cells 1 (NFATc1), and Dendrocyte expressed seven transmembrane protein (DC-STAMP) on osteoclast cells [77]. Bone resorption process involves two phases hallmarked by a resorption or Howship’s lacuna. In the first phase, the mineral components (mostly consisting of hydroxyapatite) are dissolved through acidification of lacuna mediated by action of the proteases, carbonic anhydrase II, and Vacuolar type proton pump (V-ATPase) and chloride channels. In the second phase, the organic matrix components are resorbed by lysosomal proteases (Cathepsins) and matrix metalloproteases. The degraded proteins including type 1 collagen fragments are released into extracellular space and are destroyed by fusion with TRAP enriched cytoplasmic vesicles [78]. The process of osteoclastogenesis is countered by Osteoprotegerin (OPG), which is released by osteoblasts to inhibit the Receptor Activator of nuclear kappa-B ligand (RANKL) binding [79]. Additionally, at this stage, osteoclasts release molecules called semaphorins, which inhibit osteoblast formation. This is followed by a transition or reversal stage subsequently leading to bone formation by osteoblasts. Post this reversal stage, osteoclasts express ephrinB2 on their membrane, which binds to ephrinB4 on osteoblasts for promoting osteoblastic differentiation [80]. The cellular crosstalk is not limited to the resident bone cells. Bone cells also have close communication with cells of the microenvironment. Since progenitors of bone and immune cells are shared, they express common cytokines and receptors. Activated immune cells such as dendritic cells, macrophages, and T lymphocytes produce inflammatory cytokines that stimulate bone loss through resorption [81]. Additionally, there is a synergistic crosstalk between the osteoblast precursors and endothelial cells. Angiogenesis and osteogenic differentiation are coupled events for bone regeneration. While vascularization is a key process for osteogenesis, there is also evidence that mesenchymal stem cells stimulate the recruitment, proliferation, and migration of endothelial cells while enhancing their ability to form capillary-like structures through Vascular endothelial growth factors (VEGF) production [82]. Therefore, the process of bone remodeling involves coordinated communication between the bone cells as well as microenvironment cells through soluble signaling molecules, cytokines, hormones, etc. One of these paracrine signaling factors involved are cell-secreted nanometer-sized extracellular vesicles or exosomes, which will be the focus of our further discussion.

## 3. Exosomes in Bone Tissue Microenvironment 

**Figure 2:**
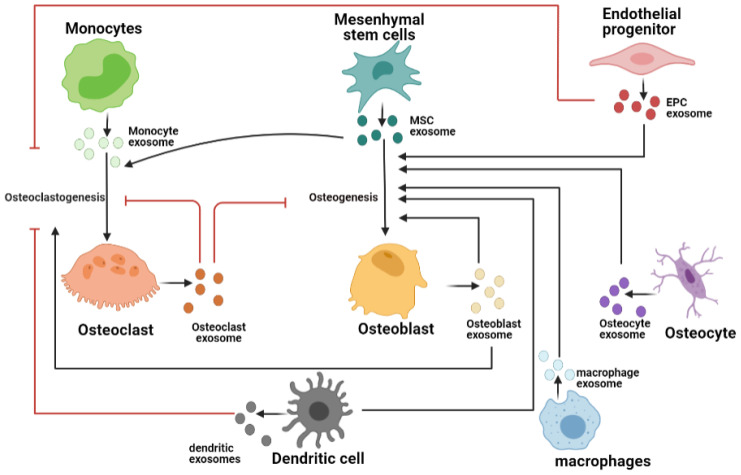
Schematic representation depicting selected role of exosomes from resident and microenvironment cells in bone remodeling: Hematopoietic precursors such as monocytes secrete exosomes, which stimulate osteoclast differentiation. Mature osteoclast-derived exosomes are enriched with RANK, which competitively inhibits osteoclastogenesis while also inhibiting osteogenesis in early stages. Mesenchymal stem cells secrete exosomes, which promote osteogenesis. MSCs and osteoblasts also secrete RANKL enriched exosomes, which stimulates osteoclastogenesis. Osteoblast-derived exosomes further promote mineralization and osteogenesis. Terminally differentiated osteocytes derived from sub population of osteoblasts promote bone formation under mechanical stress conditions. Cells in microenvironments such as endothelial cells and dendritic cells promote bone formation while inhibiting bone resorption. Macrophages also undergo polarization to stimulate osteogenesis.

While the signaling pathways have been very well studied to understand bone tissue homeostasis, there have been relatively few studies to understand the role of exosomes mediated bone remodeling [83,84]. In context to bone homeostasis, exosomes derived from resident bone cells such as osteoblasts, osteocytes, osteoclasts, and other cells [85,86,87] in microenvironment such as mesenchymal stem cells, immune cells, and endothelial cells have been demonstrated to influence bone formation, resorption (illustrated in Figure 2), and development of tumors and bone pathologies [85,88,89]. These exosomes are enriched with signaling molecules and short non-coding RNAs and proteins specific to bone remodeling [9]. Most of the earlier studies concentrating on exosomes in bone homeostasis have been performed on mesenchymal stem cells due to therapeutic potential of these cells for bone repair and remodeling in several bone-related disorders [90,91,92]. The exosomes derived from these cells demonstrated similar regenerative effects [93]. However, in the more recent few years, there has been increasing interest in exosomes derived from resident bone cells including osteoclasts, osteoblasts, and osteocytes [94]. There is substantial evidence now demonstrating the influence of resident bone cell-secreted exosome and its cargo on various signaling mechanisms and bone homeostasis [95,96]. These studies have provided us with novel insights and possible directions for discovery of early diagnostic markers for bone pathologies as well as targeting specific cargo for therapeutic treatment [97]. In parallel, there have been studies for better physiological understanding of the communication of bone cells with microenvironment cells such immune cells, endothelial cells, and stromal cells in influencing bone homeostasis [98,99]. In the coming sections, we will begin with describing exosomes derived from various resident bone cells followed by exosomes secreted by progenitor and microenvironment cells. We will also describe how tumor-derived exosomes interact with the bone cells to influence tumor progression. 

### 3.1. Osteoclast-Derived Exosomes

Osteoclasts are multinucleated bone cells derived from hematopoietic precursors such as peripheral blood/bone marrow monocytes and macrophages and are responsible for bone resorption during remodeling. Extracellular vesicles (EVs) released from osteoclasts are regulators of osteoclastogenesis. While EVs from the precursor cells stimulate osteoclast differentiation, EVs from mature osteoclasts inhibit osteoclastogenesis in the same cultures. This is attributed to competitive inhibition by Receptor activator of nuclear kinases (RANK) present in mature EVs [100]. A detailed proteomic analysis of the secretome of EVs shed by osteoclasts through mass spectrometry has revealed the presence of abundant actin associated proteins and integrin proteins in these extracellular vesicles. This can also be correlated with the fact that osteoclasts form actin rings on their membranes in resorption compartments and these may also play role in the formation of EVs when they bud off the membrane. The actin proteins have also been proposed to be responsible for maintaining integrin conformation to promote their high affinity binding with matrix at specific sites in organs and tissues [101].

Osteoclast-derived exosomes have been demonstrated to promote osteogenic differentiation of stromal cells prior to osteogenesis [102]. Contrastingly, these exosomes have also been shown to be internalized within osteoblasts via EphrinA2/EphA2 recognition to inhibit their differentiation leading to reduced bone formation [103]. Exosomes secreted by osteoclasts mediate their action on target cells through their microRNA and protein cargo. Recently, upregulation of a microRNA, miR-23a-5p, has been reported to inhibit osteogenic differentiation by regulating *RUNX2* or Yes-associated protein-1 (YAP1) mediated Metallothionein 1D Pseudogene (*MT1DP*) inhibition [104]. Thus, EV cargo could be responsible for directing specific responses in target cells. 

Apart from role in signaling mechanisms, osteoclast-derived exosomes could also be crucial for discovering potential diagnostic markers for early detection of bone disease. For instance, increased serum exosomal levels of miR-214-3p have been associated with reduced osteogenesis in elderly women as well as ovariectomized mice, which is rescued upon treatment with miR-214 inhibitor, indicating circulating levels of this microRNA as an crucial marker for bone loss [105]. This further indicates that targeting serum microRNAs could be a potential strategy for detection and follow up treatment of bone disorders.

### 3.2. Osteoblast-Derived Exosomes

Osteoblasts are the resident bone cells derived from bone marrow mesenchymal stem cells responsible for the synthesis and mineralization of bone matrix through release of collagen and glycoproteins. Mineralizing osteoblast-derived exosomes have been demonstrated to induce osteogenic differentiation of mesenchymal stromal cells via activation of Wnt signaling (inhibition of Axin1 and overexpression of beta catenin proteins), calcium signaling, and microRNA profile modulation [106]. These exosomes have been shown to even override extra cellular matrix instructions for lineage specific differentiation of stromal cells [107]. In one study, a comprehensive analysis of the secreted extra cellular vesicles demonstrated temporal changes in proteomic profiles during different stages of osteoblast mineralization. The cargo of EVs was constantly changing and there were evident changes in later stages of mineralization including reduced vesicle integrity, particle concentrations, and enrichment in proteins with specific functions in extracellular matrix organization, angiogenesis, and skeletal development. There was further an increased expression of Annexin proteins involved in calcium channeling and Wnt proteins [108]. Similar changes in proteomic profile were observed by Bilen and colleagues revealing proteins unique to both proliferating and mineralizing osteoblasts. One specific adhesion protein, Cadherin-11, was found to play a unique role in uptake of osteoblast-derived exosomes [109]. Other proteomic studies of osteoblast-derived exosomes have revealed upregulation of (Transforming growth factor beta) TGFB3, Low Density Lipoprotein Receptor-related protein 6 (LRP6), BMP-1, SMAD specific E3 Ubiquitin Protein Ligase 1 (SMURF-1) proteins [110], and the eukaryotic initiation factor 2 pathway [111] in osteogenesis. However, when these healthy osteoblasts attain a malignant phenotype such as in osteosarcoma, the proteomic profile of osteoblast-derived exosomes is modulated with expression of immunosuppressive proteins such as TGB1, alpha-fetoprotein, and heat shock proteins. They also promote regulatory T cell phenotype with significantly reduced T cell activity [112].

Osteoblast-derived EVs have also been demonstrated to stimulate osteoclast differentiation via RANKL-RANK signaling. These EVs were found to be rich in RANKL protein, which was internalized by osteoclast precursors and also caused nuclear translocation of *NFATc1*, a master transcription regulator of osteoclast differentiation [113]. In an interesting study, live cell imaging within fracture healing model of transgenic zebrafish demonstrated internalization of osteoblast-derived extracellular vesicles within osteoclasts to promote osteoclast differentiation [114] (Table 1).

Exosomes from osteoblast also regulate angiogenesis, a crucial process during bone homeostasis. They have been shown to promote angiogenic potential of endothelial cells via the VEGF/Extracellular signal-regulated kinases (ERK1/2) pathway through release of Matrix Metalloprotein2 (MMP2) [115]. This crosstalk is crucial even during pathogenesis of age-related disorders. In an interesting study, senescent osteoblast-derived exosomes were found responsible for accelerated senescence and aging of endothelial cell cultures via release of miR-139-5p. This revelation could indicate a possible mechanism through which osteoblasts regulate endothelial cell function during age-related bone disorders such as osteoporosis [37].

Osteoblast EVs are not mere regulators of intercellular bone communication but are also able to deliver anti-osteoclastic drugs and active molecules for targeted therapies. Cappariello and colleagues demonstrated that osteoblast-derived EVs can be loaded with clinically approved anti-osteoclastic drugs for malignancies (dasatinib) and osteoporosis (sodium zoledronate) with retained efficacy and functional effects even both in vitro and in vivo mouse models [116]. Future research on osteoblast secretome can reveal specific biomolecules that can be targeted for skeletal disorders.

### 3.3. Osteocyte-Derived Exosomes

Osteocytes are terminally differentiated bone cells that constitute the majority of the resident cell population in bone. While soluble factors and signaling molecules released by osteocytes have been proposed to play key role in bone homeostasis, there are currently very limited studies on osteocyte-secreted exosomes. Studies have shown that osteocyte-secreted exosomes are found to circulate in blood and also contain miRNA that may contribute to bone remodeling [117]. Osteocytes are mechanosensitive cells that respond to external stimuli through modulation of their secretome. Osteocyte-derived exosomes may promote osteogenic differentiation on exposure to mechanical strain. Exosomes from osteocytes led to osteogenic differentiation of human periodontal ligament stem cells. RNA sequencing has shown a possible mechanism through upregulation of miR-181b-5P, which inhibits Phosphatase and Tensin homolog (PTEN) and activates protein kinase B (Akt) [118]. In a similar study, osteocytes subjected to fluid sheer were able to enhance the recruitment of stromal progenitor cells to direct osteogenesis via a unique set of extracellular vesicles [119]. Morrell et al. decoded the mechanism of osteocyte mechanosensitivity through fluid sheer flow-induced calcium oscillation studies. The osteocytes developed immediate actin contractions, which were found to be regulated by smooth muscle actin through increased secretion of extracellular vesicles. Further, Lysosomal associated protein (LAMP1) was upregulated in the secretome, which further induced bone formation [120]. Osteocytes have also been reported to communicate synergistically with muscles to augment osteogenic differentiation. Muscles secrete several cytokines including myostatin for negatively regulating muscle growth and differentiation. Studies have also linked the inhibitory role of myostatin during bone formation. In one report, exosomes from myostatin-treated osteocytes inhibited osteogenic differentiation by blocking the Runx2 and Wnt pathway through miR-218 [121].

Osteocyte-derived exosomes have been correlated with development of bone pathologies. In one study, osteocytes were found to secrete mir-124-3p containing EVs, which increased under high glucose conditions (simulated for Diabetes mellitus). The mir-124-3p targeted galectin-3 in osteoblast cells, a factor specifically associated with inflammatory conditions and Diabetes [122]. This suggests a possible role of the osteocyte EV cargo in aggravated bone loss in Diabetic patients. 

There is huge scope for study of osteocyte-derived exosomes on 3D bone tissue constructs, an area that has been completely unexplored till now. Since osteocytes in vivo are embedded within heavily calcified matrix, which affects mechanosensing as well as oxygen diffusion, the current 2D in vitro models are unable to fully mimic the natural osteocyte function. Preliminary attempts to generate osteocytes 3D models have used collagen gels or biphasic calcium phosphate microbeads [123]. Future research in context to bone specific biomaterials may reveal interesting details on signaling mechanisms and help in modeling diseases. 

### 3.4. Endothelial Cell-Derived Exosomes 

Angiogenesis/neovascularization has a key role in bone microenvironment ensuring remodeling and repair. Osteogenesis is known to be closely coupled with angiogenesis during neo-bone formation. Endothelial cell-derived exosomes are able to efficiently target bone cells to stimulate bone regeneration (Table 2). In a study of distraction osteogenesis, endothelial progenitor cells (EPC)-derived exosomes were able to accelerate bone formation by stimulating angiogenesis via Raf/ERK signaling involving upregulation of miR-126 [124]. These results were supported by the induction of in vitro osteoblast differentiation of bone marrow stromal cells mediated by EPC-derived extracellular vesicles [125]. In another study, EPC were shown to promote healing and neovascularization in a mice bone fracture model through recruitment of osteoclast precursors. EPC-derived exosomes enhanced osteoclastic differentiation of bone marrow macrophages through long non-coding RNA-Metastasis-Associated lung adenocarcinoma Transcript-1(MALAT1), which negatively regulated miR-124 [126].

EPC also prevent osteoporosis through promotion of osteogenesis or inhibition of osteoclastogenesis. EC-derived exosomes were found to be internalized more efficiently by bone marrow macrophages than osteoblasts or mesenchymal stem cells. Further, these exosomes are able to inhibit osteoclasts through upregulation of miR-155 and, thus, shown to play role in preventing osteoporosis in mice models [127]. In a bioinformatics study, it was noticed that EPC-derived EVs were able to inhibit mouse glucocorticoid-induced osteoporosis through a ferroptotic pathway in osteoblasts [128]. Endothelial cell-derived exosome can prevent osteonecrosis through promotion of osteogenesis via miR-27-a [129]. This confirms the role of endothelial cell-derived exosomes in bone repair and as a possible therapy for bone resorption disorders.

### 3.5. Immune Cell-Derived Exosom

Immune cells in the bone microenvironment have demonstrated a specific activating or inhibiting response towards osteoblasts and osteoclasts through the release of cytokines and paracrine factors (Table 2). Dendritic cell-derived EVs have been shown to induce osteogenesis [130]. A proposed mechanism involves role of Hippo signaling and exosomal miR-335 in induction of bone regeneration through Large tongue suppressor kinase 1 [131]. Dendritic cell-derived exosomes contain immunoregulatory cargo (TGFB1 and IL-10) that is selectively released upon inflammation to promote regulatory T cell recruitment, which, ultimately, inhibits osteoclasts and bone loss [132]. Exosomes derived from unpolarized M0, polarized M1 (pro-inflammatory phenotype), and M2 (anti-inflammatory phenotype) macrophages have shown distinct osteogenic lineage specification of bone marrow-derived mesenchymal stem cells [133]. A further analysis into the macrophage-derived exosomal microRNA-5106 revealed that it can induce osteoblast differentiation by Salt inducible kinase 2 and 3 genes [134]. Understanding the immune responses in bone diseases is essential for drug discovery and development. Moreover, co-cultures with immune cells on both 2D and 3D bone platforms can help in generating more physiologically relevant models for bone disease. Future research on modulation of exosome cargo in response to immune cells will contribute to a greater understanding of the crosstalk in bone microenvironment.

### 3.6. Exosomes from Mesenchymal Stem Cells

Mesenchymal stem cells (MSC) are the precursors for osteoblast differentiation. MSC have been widely reported to promote bone repair and regeneration through their paracrine action. Secretome analysis of MSC has revealed that this role is mediated through modulation of the microRNA profile in the released exosomes [135]. While exosomes at early stages of osteogenic differentiation promote the expression of early osteogenic markers, at later stages they are responsible for increased migration and proliferation of MSC [136]. Furthermore, the regenerative action of MSC-derived exosomes is also age dependent where younger donors have enhanced osteogenic capacity [137]. With increasing age, there is modulation in the microRNA profile [138]. For instance, microarray analysis of bone marrow MSC-derived exosomes revealed that miR-128-3p was significantly upregulated in aged mice with bone fractures and targeted Smad5. An experimental series of gain and loss of function assays verified that miR-128-3p is a suppressor of fracture healing [139]. Similarly, miR-31a-p, responsible for osteoclastogenesis, is significantly upregulated in aging patients [140] Therefore targeting miRNA through specific antogimirs or knockdown may be an approach for enhanced healing in elderly patients.

Several reports have indicated that MSC-derived exosomes could repair bone fractures through enhanced osteogenesis as well as angiogenesis [63,64]. Zhang et al., reported that bone marrow MSC-derived exosome transplantation into a rat model of bone fracture-induced osteogenesis, angiogenesis, and healing. Furthermore, in vitro, these exosomes could be internalized by osteoblast precursor cells as well as human umbilical cord vein endothelial and activate BMP-2/SMAD-1/RUNX2 pathways that seems to play an important role in the induced effects [141]. Exosomes have also been reported to enhance osteoblast proliferation via the Mitogen-activated protein kinase (MAPK) pathway [142]. The role of angiogenesis was further reported to prevent osteonecrosis due to local ischemia at site of bone damage through PI3K/Akt signaling. The suppression of osteonecrosis is mediated by action of miR-122-p by targeting Sprouty (SPRY2) and mir-34c sponged by the tumor-related long non-coding RNA MALAT-1 to target Special AT-rich sequence binding protein 2 3′UTR (SATB2-3′UTR) [143,144]. It has also been shown that MALAT-1 sponges miR-143 during osteogenic differentiation while promoting Osterix expression [144,145]. The regenerative capacity of exosomes could further be enhanced through pre-conditioning of mesenchymal stem cells. Liu et al. reported that hypoxia-treated bone marrow MSC could enhance bone fracture repair through miR-126 by activating of the SPRED1/Ras/Erk signaling pathway [146]. It was further shown that MSC-derived exosomes carry a mutant HIF-1a, which is responsible for neovascularization and osteogenesis in critical sized bone defects [147]. In another report, pre-conditioning of MSCs with low doses of dimethyloxaloylglycine (DMOG) led to enhanced angiogenic effect through the Akt/mTOR pathway and subsequent osteogenesis [148]. Lu et al. demonstrated that priming of mesenchymal stem cells with TNF-a promotes bone repair capacity of exosomes by mimicking the acute inflammatory stage of bone injury [149].

Apart from bone fracture healing, MSC-derived exosomes have been found to play protective role in alleviation of osteoarthritis (OA) through bone and cartilage healing [150]. MSC-exosomes inhibit catabolic and inflammatory markers as well as play a chondroprotective role in osteoarthritis models [90,150]. The anti-inflammatory response was found to be mediated by bone marrow MSC-derived miR-192-5p and miR-127-3p [151,152], which have protective function towards chondrocytes. They also display pain relieving properties by abrogating nerve invasion into the joint [153,154]. These exosomes have demonstrated immunoregulatory role at different stages of osteogenic differentiation. Exosomes secreted by MSC during osteogenesis have the potential to be taken up by macrophages to direct their polarization from the M1 to M2 phenotype. Alleviation of OA is mediated through macrophage polarization with inhibition of M1 phenotype while promotion of M2 phenotype. Macrophage polarization can be further promoted by pre-treatment of MSC with TNF-a, which upregulates miR-1260 to target Wnt5a and inhibits osteoclast activity [155].

Exosomes could also potentially be used for treatment of osteosarcoma by direct intravenous injection. Studies have shown that exosomes have similar regenerative abilities as MSC transplantation. Since these exosomes are immune privileged, this could potentially be a cell-free therapy for cancer patients without risk of any teratoma formation. They can also be loaded with chemotherapeutic drugs such as Doxorubicin for enhanced anti-tumor activity [156].

While MSC-exosomes have a healing response towards bone disorders, they are also responsible for tumor progression and invasion into the bone. MSCs have been shown to be recruited to the tumor site and modulate their microRNA profile to mediate metastasis. Zhao et al. demonstrated that bone marrow MSC-derived lncRNA plasmacytoma Variant Translocation 1(PVT-1) is transported to the osteosarcoma site where it binds directly with the oncogenic protein ERG while sponging miR-183-5p to mediate metastasis [157]. Similarly, miR-183-5p targets Programmed cell death protein-4 (PDCD4) through the ERK1/2 pathway for promoting the invasion of osteosarcoma [83]. Furthermore, exosomes from bone marrow MSC promote osteosarcoma cell proliferation and migration by activating autophagy. Huang et al., demonstrated that silencing autophagy-related gene 5 prevented the tumor progression [158]. There is also indication that exosomes from bone marrow stromal cells are responsible for progression of multiple myeloma through miR-10a and 16 [159].

Thus, MSC-derived exosomes have been demonstrated to both promote [85,86,87,88,89,90,91,92,93,94,95,96,97] as well as inhibit osteogenic differentiation [91,98,99,100,101,102,103,104,105,106,107,108,109,110,111,112,113,114,115,116,117] through modulation of microRNA profile as listed in Table 3.

### 3.7. Tumor-Derived Exosomes

Several tumor cells such as those with prostrate, lung, breast, and oral origin are highly prone to bone invasion through crosstalk with osteoblast and osteoclast cells. This initial communication of tumor cells with bone microenvironment is mediated through paracrine secretion by tumors and is crucial for pre-metastatic niche formation. There is substantial evidence directing a role of extracellular vesicles and specifically exosomes in tumor progression via bone invasion and metastasis. Tumor-derived exosomes have demonstrated to interact with osteoclasts for promoting bone invasion and osteoclastogenesis [160,161]. This ultimately leads to an imbalanced bone homeostasis with increased bone resorption and reduced cortical bone volume/osteogenesis. Some of the key pathways targeted by tumor-derived exosomes include PI3/Akt, MAPK, and calcium signaling, which are associated with increased osteoblast proliferation, differentiation, and inhibition of osteoporosis under normal conditions [162]. Multiple myeloma is one such malignant bone disease hallmarked by an osteolytic response. Progression of multiple myeloma is mostly dependent on the cellular crosstalk between tumor cells and bone marrow microenvironment. Exosomes from multiple myeloma cells have been shown to promote osteoclastogenesis and inhibit osteogenic differentiation [163]. These exosomes are also packed with long-coding RNA, *RUNX2-AS1*, which are transmitted to MSCs, thus repressing their osteogenic potential [164].

In osteosarcoma, which is a primary bone tumor, the released exosomes were found enriched in miR-501-p, which aggravated bone loss through the PTEN/PI3/Akt pathway [165]. Raimondi et al. demonstrated that osteosarcoma-derived exosomes can also trigger a survival pathway by inducing differentiation and migration of pre-osteoclasts through CXCR4 expression [166]. The group has recently reported that these exosomes act via the Epidermal growth factor receptor (EGFR) pathway through expression of the amphiregulin, an EGFR ligand [167]. Amphiregulin expression has also been reported in non-small cell lung carcinoma (NSCLC)-derived exosomes to induce osteoclast differentiation [168].

Several studies have reported specific microRNA and protein profiles of the tumor-derived exosomes involved in bone remodeling. A study of the exosomal cargo of lung adenocarcinomas associated with bone metastasis has shown promotion of osteoclast differentiation via upregulation of miR-21 [169] and miR-214 [170]. The role of miR-214 and miR-21 in tumor-derived exosomes in bone invasion has been extensively reported in other cancers as well. For instance, prostate cancers can invade bone through downregulation of miR-214 [161] and upregulation of COL1A targeting miR-92a-1-p [171] and Cavin-1 [172]. Similarly, breast cancer-derived exosomes are associated with reconstructing the bone microenvironment. Studies have shown that the breast cancer cells transfer miR-21 to osteoclasts [103] and miR-20a-5p to bone marrow macrophages, which further promotes proliferation and differentiation of osteoclasts and ultimately invasion of the tumor cells [173] Recently, it was shown that these exosomes release a protein, L-plastin, to promote osteolysis [174].

Another pathway for tumor-induced osteoclastogenesis was recently described. Tumor-derived exosomes, namely from oral squamous carcinoma cells, target osteoclast precursor cells to induce osteoclastogenesis, which was shown to be resistant to RANKL and TGFB1 inhibitors, which normally block this process [175]. Interestingly, in the same cells and experimental setup, Cannibidol interfered with osteoclastogenesis by a mechanism independent of NFATc1 expression, a master transcription factor for this process; accordingly, this drug did not affect RANKL-induced osteoclastogenesis of precursor cells [176].

Apart from targeting osteoclasts, exosomes from tumor cells, namely, prostate cancer cells, can also induce osteogenic differentiation of human mesenchymal stem cells in vitro and promote osteoblastic metastasis in the bone microenvironment in vivo via hsa-miR-940 [176].

There have been recent efforts made to prevent bone metastasis and tumor progression. Faict and colleagues demonstrated in multiple myeloma cells that blocking the exosome secretion from the tumor cells resulted in strong anti-tumor response with increased cortical bone volume, indicating a potential therapeutic target for the disease [177]. Further, prostate tumor cells communicate with bone marrow myeloid cells for promoting metastasis through a cholesterol dependent mechanism. Reducing the cholesterol in myeloid cells can prevent uptake of extracellular vesicles, thus reducing osteoclastogenesis [178]. These authors suggested the potential for diagnostic and therapeutic approaches to prevent metastasis by modulating metabolic factors relevant in pre-metastatic niche formation.

## 4. Exosomes in Bone Tissue Engineering

**Figure 3:**
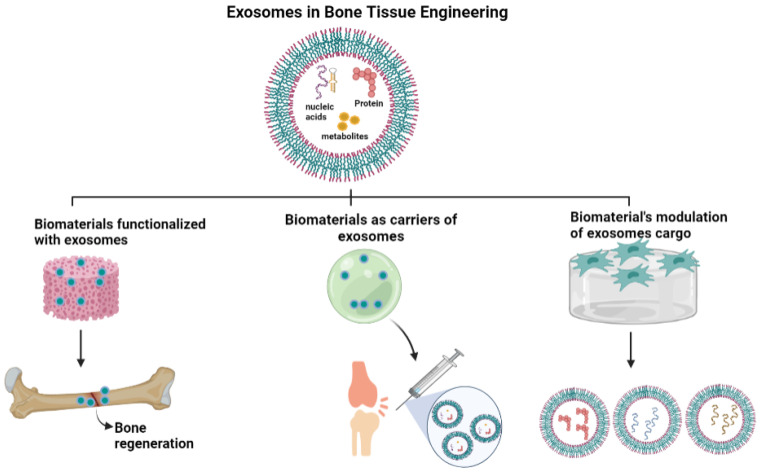
Exosomes in Bone Tissue Engineering. Studies of exosomes in Bone Tissue Engineering are grouped into 3 broad categories-1. Biomaterials functionalized with exosomes 2. Biomaterials as carriers of exosomes 3. Modulation of exosome cargo by biomaterials.

Bone tissue engineering has been a widely studied field wherein several biomaterials are investigated as suitable bone graft substitutes for clinical transplantation [178]. The scaffolds are tested for physiochemical properties such as mechanical and tensile strength, porosity, corrosion resistance, wear and fatigue resistance, biodegradation, and cytocompatibility [179,180]. There have been lot of biological studies directed towards functionalizing the bone-specific scaffolds though embedding cells and checking their viability, proliferation, and differentiation ability [181,182]. There are also studies performed on various signaling pathways being modulated when cells interact with biomaterials. Recently, there has been increasing interest in analysis of secretome of cells embedded within biomaterials including release of growth factors, signaling molecules, and extracellular vesicles to better understand the molecular mechanisms involved during this interaction [183,184,185]. One of the key extracellular vesicles are exosomes that have a role in mediating regenerative effects of the cells. Exosomes are a relatively unexplored area in bone tissue engineering requiring further in-depth molecular exploration [186]. In the following sections, we have grouped the studies on exosomes into three broad categories (Figure 3). The first area focuses on functionalization of biomaterials with exosomes instead of cells for cell-free therapy to mediate enhanced repair of bone defects and disorders [187]. This is crucial, especially in treatment of critical-sized or large bone defects requiring a scaffold for gap filling. This field has diverse clinical applications and exploits the reparative potential of exosomes in adjunct to the biomaterials. The second area focuses on using biomaterials as a delivery agent or carrier for exosomes [188]. This field encompasses pre-conditioning or engineering exosomes before delivery to the target defect site for specific applications. It exploits the regenerative potential of exosomes and their osteoinductive cargo, specifically, for bone diseases and small bone defects; although, they are now being explored for critical size bone defects too [189,190]. The third category focuses on the potential of biomaterials to modulate exosome cargo and secretion of the interacting cells, hastening the local regenerative events [191].

This is an upcoming exploratory field encompassing a completely innovative paradigm in bone regenerative strategies, with potential for the discovery of biomolecules in exosomes for targeted therapy strategies, with potential for the discovery of biomolecules in exosomes for targeted therapy.

### 4.1. Exosome-Functionalized Bone Biomaterials

**Figure 4:**
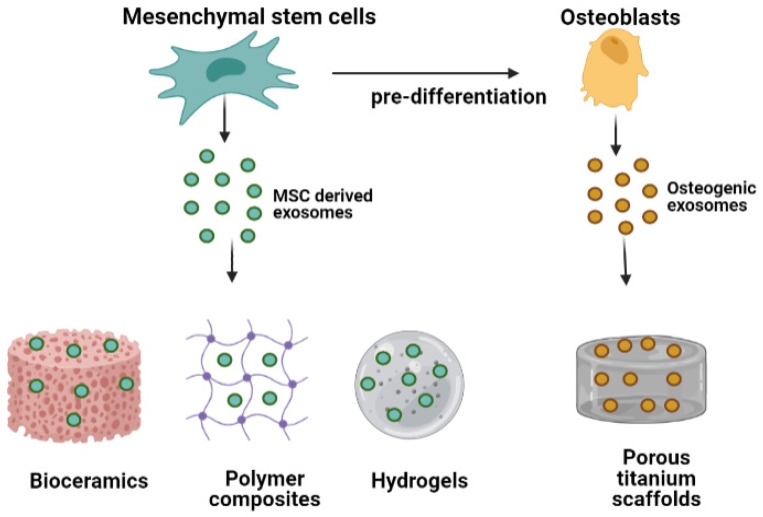
Schematic representation of exosome conjugation strategies over bone specific biomaterials. Exosomes derived from both mesenchymal stem cells as well as osteogenically pre-differentiated MSCs have been immobilized over bioceramics, polymer composites, hydrogels (as carriers), and titanium scaffolds for functionalization.

Traditional bone research has focused on embedding mesenchymal stem cells on bone specific biomaterials to mediate enhanced repair and regeneration [192,193]. However, the application of mesenchymal stem cells has been limited for clinical purposes due to concerns of safety, ethical issues, limited supply of source tissue, and risk of teratoma formation in both autologous and allogenic transplantation cases [194,195]. Cell therapy is also a challenge for larger bone defects, which are characterized by an ischemic microenvironment leading to poor survival of transplanted cells and competition with endogenous cells for oxygen and nutrients [196]. Recently, with the emergence of cell-free therapies, researchers are now exploring the role of the secretome of stem cells in inducing similar regenerative effects. Exosomes derived from MSC have demonstrated similar regenerative effects as their cellular counterpart [197]. Furthermore, these exosomes are immune privileged and contain lesser membrane proteins (such as the major histocompatibility complex) and, thus, safe for clinical applications. Their cargo, including miRNAs and proteins, is protected by a lipid bilayer and they interact with target cells through endocytosis. Recent evidence suggests that MSC-derived exosomes are capable of guiding osteoinduction. Exosome delivery would also be potentially advantageous in critical size bone defects where MSC survival post engraftment is poor. However, injection of exosome alone directly at the tissue site is inefficient in rescuing osteoporotic phenotype and larger bone defects [198]. Therefore, exosomes need to be immobilized on biomaterial scaffolds for structural support and better functionalization. Exosome conjugation on biomaterials has been studied on bioceramics, polymers, composite scaffolds, and bioactive metals for enhanced bone regeneration in several disease models (Figure 4). Strategies currently exploited for EV functionalization on biomaterials include exploitation of electrostatic interactions between negatively charged exosomes and positively charged materials and adhesion with extracellular matrix proteins such collagen, fibronectin, etc. [199].

#### 4.1.1. Exosome-Conjugated Bioceramics

Direct injection of bone marrow mesenchymal stem cells has been widely investigated for clinical application due to their superior osteogenic differentiation potential. However, local injection of cells at the damage site is often not suitable for large-sized bone defects due to insufficient mechanical support, pressure, and load bearing capacity [200]. To overcome this limitation, bioceramics are a preferred scaffold choice for implanting these cells due to their superior mechanical strength, microporosity, biocompatibility, and biodegradability [201]. Bioceramics can be resorbable such as β-tricalcium phosphates, bioactive such as glass and hydroxyapatite glass ceramics, or non-resorbable/inert such as zirconia and alumina. While MSCs have been studied clinically with bioceramics for bone regeneration, there is a growing interest in conjugation of MSC-derived exosomes on to the hard scaffolds for improved bioactivity of scaffolds due to their having similar regenerative properties as MSCs. In one of the early reports, conjugating exosomes on ceramics, it was shown that the loading of secretion factors from MSCs on to hydroxyapatite/tricalcium phosphate before implantation could induce new bone formation as confirmed through osteoid matrix visualization in H&E staining [202]. Qi et al. demonstrated that MSC-derived exosomes could repair critical-sized bone defects through enhanced osteoblast differentiation and angiogenesis over tricalcium phosphate (TCP) scaffolds as compared to pure scaffolds [203]. It was further demonstrated that the conjugated exosomes from TCP were released and internalized into BM-MSCs, which further promoted their migration and osteogenic induction compared to pure scaffolds in a PI3K/Akt pathway-dependent manner [204]. Nowadays, mineral doping of ceramics is performed to enhance their bioactivity. Previous studies have reported increased osteogenic commitment of MSC through substitution of trace elements or mineral doping in biomaterials. Therefore, the presence of mineral fillers in scaffolds is being studied to improve the uptake and retention of exosomes. These conjugated exosomes are functional and release from scaffolds to interact with mesenchymal stem cells to further stimulate osteogenic differentiation [205]. In a recent study, strontium-substituted calcium silicates boosted osteogenic differentiation as well as angiogenic potential of exosomes in both zebrafish and rat femur defect models. The exosome cargo was enriched in pro-angiogenic miR-146a, which targeted Smad4 (involved in TGF-b signaling) and NF2 proteins (involved in Hippo signaling), thus inhibiting their expression in Human Umbilical vein endothelial cells (HUVEC) cells. In vivo studies validated neovascularization and bone tissue regeneration [206]. Bioceramics have excellent clinical potential demonstrating osteogenic functionalizing with exosomes. Improved fabrication strategies can further be explored to conjugate exosomes and perform study release dynamic under in vivo conditions.

#### 4.1.2. Exosome Conjugated Polymers

Synthetic biodegradable polymer systems have been investigated as efficient delivery platforms for exosomes due to their tunable release kinetics. Exosomes derived from adipose mesenchymal stem cells were immobilized onto poly (lactic-co-glycolic acid) (PLGA) scaffolds with polydopamine coating. The exosomes were shown to release consistently in vitro, and promote bone regeneration in vivo. This was linked to its capacity for promoting MSC homing to the site of bone injury [207]. Co-polymer systems such as PLGA-polyethylene glycol triblocks have also been explored as delivery platforms for controlled release of exosomes with osteogenic capacity comparable to exogenous delivery [208]. Another popular choice of bone specific biomaterial is Polycaprolactone, which is an FDA approved resorbable biomaterial. However, the clinical applications of PCL are limited due to lack of bioactivity. Exosomes from MSC have been instrumental in functionalizing these scaffolds to act as barrier membrane for guided osteogenesis [209]. Polymer composites have comparable elastic modulus with bone but require improved bioactivity. Further studies on exosome conjugation on polymer scaffolds possess huge scope in functionalization.

#### 4.1.3. Exosome Conjugated Metals

Metals have been the oldest bone graft choice for implantation due to their mechanical properties [210]. Among metals, titanium and its alloys are widely preferred for bone tissue engineering due to desirable properties such as biocompatibility, non-toxicity, optimal porosity for cell migration and proliferation, high mechanical strength, and corrosion resistance. Moreover, they have been shown to promote osseointegration and osteogenesis [211,212]. Recent research is being focused to generate bioactive metals to improve their biological functionality. In one report, titanium scaffolds were generated by selective laser sintering 3D printing method. MSC were osteogenically pre-differentiated for 10–15 days before immobilization of their secreted exosomes on 3D-printed titanium scaffolds. Exosome adsorption was performed by coating the scaffold surface with positively charged poly-lysine to mediate charge interactions. These pre-differentiated exosomes had comparable osteoinductive effects as MSC loaded on the same scaffolds. RNA sequencing data of these exosome suggested that there is upregulation in specific miRNA signature to activate MAPK and PI3K/Akt signaling. Highly expressed microRNAs included osteogenic differentiation specific miR-146a-5p, miR-503-5p, miR-483-3p, and miR-129-5p and negatively expressed miRNAs included anti-osteogenic microRNAs such as miR-32-5p, miR-133a-3p, and miR-204-5p [58]. Another proposed mechanism through which pre-differentiated exosomes stimulate osteogenesis is binding with extracellular matrix (ECM) proteins such as collagen type 1 and fibronectin [213]. Additionally, there have been efforts to generate porous Titanium-based alloys to match the elastic modulus of bone. Wu et al. generated porous Ti6Al4V by electron beam melting 3D printing and doped them with Schwann cell-derived exosomes to demonstrate their capacity for bone formation [214]. In another attempt to improve the functionality of titanium implants, exosomes from BMP2 stimulated macrophages were integrated into titanium nanotubes. The integration led to significant enhancement in osteogenic differentiation and uptake by MSC [215]. The study also gave novel insights into possible ways of replacing BMP2 treatment that can lead to side effects such as ectopic bone formation.

### 4.2. Biomaterials as Carriers of Osteogenic Exosomes

Exosomes are an attractive alternative to cell-based transplantations to avoid safety and ethical concerns. They are also excellent osteoinductive agents. However, exosome delivery alone is met with several challenges including poor retention and lower therapeutic concentrations at the defect site. This is caused mainly due to rapid release of the exosomes to non-target sites and clearance from the reticuloendothelial system. Therefore, carriers for exosomes are required for sustained release and controlled local doses [198]. Hydrogels are the preferred carrier material for embedding exosomes and delivering them to bone defect sites due to their biocompatibility. Hydrogels have been popular for delivery of growth factors, proteins, drugs, and stem cells in target tissues due to their capability of controlling the release kinetics [199]. Exosomes embedded in hydrogels have been shown to release in a controlled and slow manner to promote bone regeneration. Moreover, hydrogel biomaterials were demonstrated to completely absorb at the defect site while stimulating osteogenic induction and enhancing bone volume [91]. Recently, several novel hydrogel composites were synthesized as exosome carriers for enhanced bone regeneration. Yang et al. fabricated an injectable hydrogel consisting of Hydroxyapatite embedded in cross-linked hyaluronic acid and alginate as a carrier of MSC-derived exosomes for efficient osteoblast differentiation [91]. In another report, a novel hydrogel composite consisting of coralline hydroxyapatite (CHA), silk fibroin (SF)/glycol chitosan (GCS), and difunctionalized polyethylene glycol proved to be an excellent graft material [216]. The hydrogel displayed self-healing properties due to formation of a stable imine bond and had good mechanical resistance. The authors further demonstrated that the exosome-containing hydrogel could enhance healing in a model of rat bone defect by promoting infiltration of osteoblasts, chondrocytes, stimulating angiogenesis and deposition of Bone morphogenic protein (BMP2). In another study, dental pulp-derived exosomes were integrated into chitosan hydrogels for relieving periodontitis, which is caused due to high level of inflammatory macrophages. The study demonstrated that pro-inflammatory macrophages were converted to anti-inflammatory macrophages with an association of miR-1246 [217]. A possible future direction is the isolation of exosomes enriched with certain microRNAs that are highly expressed during osteogenic differentiation. Shen et al. isolated exosomes from miR375 overexpressing mesenchymal stem cells and embedded them into hydrogels for enhanced bone regeneration. Another potential area is improvement of encapsulation efficiency for prolonged delivery of osteogenic exosomes. Huang et al. generated engineered extracellular vesicles by tethering them on to photocrosslinked alginate hydrogels with RGD motifs. They demonstrated that EVs could be encapsulated for up to 7 days without alteration of functional integrity while also stimulating significant bone regeneration in calvarial defect models [218].

### 4.3. Role of Biomaterials in Influencing Exosomal Cargo

While there have been recent studies on functionalization of biomaterials incorporating osteogenic exosomes, there is very little known about modulation of the secretome of cells in response to different biomaterials. Recent reports suggest that this type of biomaterial may influence the fate of cell-secreted exosomes in promoting or inhibiting osteogenesis. In one study, magnetic nanoparticle-infused hydroxyapatite scaffolds were used to study osteoporosis and it was found the scaffold promoted osteoblast differentiation through modulation of osteoclast exosome cargo including reduction in ubiquitin, ATP, and reactive oxygen species [219]. In another study, exosomes from the macrophages treated with Titanium particles inhibited osteogenic differentiation. RNA sequencing and gene ontology studies have revealed the role of two long non-coding RNAs—NONMMUT000375.2 and NONMMUT071578.2—in suppressing osteogenesis through regulation of genes Bcl2, Wnt11, TGF-β, and Pdk1 [220]. There are also recent indications that the doping of biomaterials with certain trace elements has been shown to influence the exosome cargo of mesenchymal stem cells towards enhanced angiogenic potential. In one report, lithium-containing bioactive glass ceramic promoted expression of proangiogenic miR-130a in bone marrow mesenchymal stem cells and activated Wnt, Wnt/β-catenin, AKT, and NF-Κb. The chemical cues from the biomaterial further influenced the crosstalk of stromal cells with endothelial cells during bone regeneration [221]. There is also evidence that strontium-containing biomaterials stimulate pro-angiogenic miR-146a secretion in exosomes from Bone marrow MSC leading to neovascularization [206]. There are very limited studies to conclude the specific influence of different types of biomaterials on the modulation of exosome cargo of cells. However, studies on cargo analysis of exosomes over biomaterials bring about an innovative paradigm encompassing highly specific molecular targets in bone regenerative strategies.

## 5. Future Scope and Challenges

Exosomes from various types of bone cells play a crucial role as signaling molecules in bone remodeling as well as in the progression of bone disorders [222,223]. Exosomal cargo includes proteins, small RNAs such as microRNA, long non-coding RNAs whose expression is augmented during osteomodulation [224]. Understanding the modulation of exosome-derived cargo could be a useful tool for diagnosis as well as targeting these molecules for therapeutic purposes [225,226,227,228,229]. Exosomes could in future be used as a liquid biopsy for detection of specific markers in early stages of bone disease progression [230,231]. Today, exosomes are being conceived as acellular therapies for bone regeneration in several bone disorders and age-related defects [45,127,232,233]. They possess excellent potential for off-the-shelf transplantations due to their comparable regenerative effects with cellular therapy [234,235]. Exosomes are immune privileged and, thus, there are minimal safety and ethical issues involved with their clinical application [236,237]. Moreover, due to their size, they possess the capability to enter blood capillaries, which is a restriction for MSCs. Due to this reason, exosomes are slowly gaining interest in the field of bone tissue engineering for being used as substitutes for cell seeding [238,239]. Reports have suggested that integration of exosomes on bone specific biomaterials has contributed significantly to enhanced functionality and differentiation [240,241,242]. These exosomes have been shown to release gradually from the scaffolds and are internalized in target cells for mediating their effects [208]. Scientists are now exploring several techniques for efficient integration of exosomes on scaffolds as a replacement for cells [243,244]. Recent studies are focusing on loading exosomes with specific drugs [245,246,247] or using exosomes as vectors for production of gene-activated exosomes [248,249]. Moreover, their therapeutic potential for carrying drugs is also indicated by the fact that exosomes can cross the blood–brain barrier, which restricts passage of large drug molecules [250,251]. There is also research being performed on artificial synthesis of biomimetic exosomes as a drug delivery system to specifically target inflamed or bone disorder sites [252,253,254]. The engineered exosomes are further being explored as carriers of antagomirs to target specific microRNAs for improved osteogenesis in bone disorders [255,256,257].

Currently, extracellular vesicle research faces challenges in clinical translation including large scale production and scale up from the originating cells [258,259]. Moreover, there are no defined standards on several clinical parameters such as production, processing, purification, or formulation as well as safety and purity testing for endotoxins and viruses [49,260]. Lack of consensus on established conditions for clinical use has led to heterogeneity of EVs depending on the cell source and non-reproducibility of desired effects [172,261]. Moreover, some of the limitations of cell therapy such as longer patient waiting time due to cell expansion and exorbitant cost, are still applicable to exosome use. Other key parameters that could influence the desired effects include bioavailability and concentration of exosomes to be delivered for achieving the desired therapeutic effects [262,263]. Extracellular vesicles are rapidly cleared from the circulating system and require carriers or scaffolds for sustained release to the delivered site. Finally, extensive standardization of in vivo biodistribution studies are required to check migration and accumulation of EVs in non-target organs, which could lead to off-target side effects [264,265].

## 6. Conclusions

Exosomes regulate bone remodeling process by mediating crosstalk between resident bone cells and interacting microenvironment. They have enormous potential in the field of bone tissue engineering as an acellular substitute on bone grafts. They act as excellent osteoinductive agents. Despite existing challenges in their clinical translation, they hold promising potential for regeneration and diagnosis of bone diseases and disorders. Nevertheless, it is crucial to study detailed signaling mechanisms and their cargo modulation on various biomaterials and, therefore, future studies are encouraged in this niche field.

## Figures and Tables

**Table 1:** Exosome cargo derived from bone cells and their role in bone remodeling.

Bone Cell Source	Exosome Cargo	Role in Bone Remodeling	Reference
Osteoclast	MiR-214	Inhibits osteogenesis	[114]
Osteoclast	MiR-23-a-5p	Inhibits osteogenesis	[25]
Osteoblast	Annexin	Induces osteogenesis, calcium channeling, Activation of Wnt proteins	[29]
Osteoblast	Cadherin-11	Uptake of exosomes	[30]
Osteoblast	TGFB3, LRP6, BMP-1, SMURF-1 proteins	Induce osteogenesis	[31]
Osteoblast	Eukaryotic initiation factor 2	Induces osteogenesis	[32]
Osteoblast	Matrix metalloprotein2	Induces Angiogenesis	[36]
Osteocyte	miR-218	Inhibits osteogenesis with myostatin treatment	[43]
Osteocyte	MiR-181-b-5p	Induces osteogenesis	[40]
Osteocyte	LAMP-1	Induces osteogenesis under sheer stress	[42]
Osteocyte	Mir-124-3p	Diabetes mellitus associated bone pathologies	[44]

**Table 2:** Exosome cargo derived from bone microenvironment cells and their role in bone remodeling.

Bone Microenvironment Cell Source	Exosome Cargo	Role in Bone Remodeling	Reference
Endothelial cells	Mir-155	Inhibits osteoclast differentiation	[49]
Endothelial cells	miR-126	Induces Osteogenesis	[45]
Endothelial cells	miR-27-a	Induces Osteogenesis	[50]
Dendritic cells	miR-335	Induces Osteogenesis	[53]
Dendritic cells	TGFB1 and IL-10	Inhibits osteoclasts	[53]
Macrophages	MiR-5106	Induces osteogenesis	[56]

**Table 3:** MSC-derived exosome miRNA promoting and inhibiting osteogenic differentiation.

**MSC Derived microRNA Promoting Osteogenic Differentiation**		
**Tissue Source**	**microRNA**	**References**
Human Bone marrow	MiR-15-b	[86]
Mouse Bone Marrow	MiR-25	[87]
Human Bone marrow	MiR-101	[88]
Human Bone marrow	22-3p	[89]
Human Bone marrow	935	[90]
Human Bone marrow	664-a-5p	[91]
Human Adipose	130a-3p	[92]
Human Bone marrow	30-a-5p	[93]
Human Bone marrow	133a-3p	[94]
Rabbit Bone marrow	122-5p	[67]
Rat Bone marrow	128-3p	[61]
Human Adipose	MiR-375	[95]
Human Bone marrow	200-c	[96]
Human Bone marrow	373	[97]
Mouse Bone marrow	29-a	[98]
Human Bone marrow, Human Amniotic fluid	21	[115]
**MSC-derived microRNA inhibiting osteogenic differentiation**		
**Tissue source**	**microRNA**	**References**
Human Bone marrow	Mir-29-b-3p	[99]
Human Periodontal ligament cells	MiR-23-b	[100]
Human Adipose	MiR-218	[101]
Mouse Adipose	223	[102]
Mouse Bone marrow	206	[103]
Mouse Bone marrow	145-a	[104]
Human Bone marrow	124	[105]
Rat Bone marrow	103	[106]
Human Bone marrow	144-3p	[107]
Mouse Adipose	26-a	[108]
Rat Bone marrow	MiR-31	[109]
Human Bone marrow	23-a	[110,111]
Human Bone marrow	125-b	[112]
Human Bone marrow	370-p	[113]
Human Bone marrow	221-5p	[114,135]
Human Bone marrow	143	[69]
Human bone fragments	135-a-5p	[115]
Rat Bone marrow	205	[116]
Mouse Bone marrow	339	[117]
Human Adipose tissue	130-a-3p	[92]
Rat Bone marrow	214	[77]

## Data Availability

Not applicable.
